# Versatile synthesis of end-reactive polyrotaxanes applicable to fabrication of supramolecular biomaterials

**DOI:** 10.3762/bjoc.12.287

**Published:** 2016-12-28

**Authors:** Atsushi Tamura, Asato Tonegawa, Yoshinori Arisaka, Nobuhiko Yui

**Affiliations:** 1 Department of Organic Biomaterials, Institute of Biomaterials and Bioengineering, Tokyo Medical and Dental University, 2-3-10 Kanda-Surugadai, Chiyoda, Tokyo 101-0062, Japan

**Keywords:** azide group, biomaterials, click chemistry, cyclodextrin, polyrotaxane

## Abstract

Cyclodextrin (CD)-threaded polyrotaxanes (PRXs) with reactive functional groups at the terminals of the axle polymers are attractive candidates for the design of supramolecular materials. Herein, we describe a novel and simple synthetic method for end-reactive PRXs using bis(2-amino-3-phenylpropyl) poly(ethylene glycol) (PEG-Ph-NH_2_) as an axle polymer and commercially available 4-substituted benzoic acids as capping reagents. The terminal 2-amino-3-phenylpropyl groups of PEG-Ph-NH_2_ block the dethreading of the α-CDs after capping with 4-substituted benzoic acids. By this method, two series of azide group-terminated polyrotaxanes (benzylazide: PRX-Bn-N_3,_ phenylazide: PRX-Ph-N_3,_) were synthesized for functionalization via click reactions. The PRX-Bn-N_3_ and PRX-Ph-N_3_ reacted quickly and efficiently with *p*-(*tert*-butyl)phenylacetylene via copper-catalyzed click reactions. Additionally, the terminal azide groups of the PRX-Bn-N_3_ could be modified with dibenzylcyclooctyne (DBCO)-conjugated fluorescent molecules via a copper-free click reaction; this fluorescently labeled PRX was utilized for intracellular fluorescence imaging. The method of synthesizing end-reactive PRXs described herein is simple and versatile for the design of diverse functional PRXs and can be applied to the fabrication of PRX-based supramolecular biomaterials.

## Introduction

Polyrotaxanes (PRXs) are a class of interlocked polymers that consist of an inclusion complex of cyclodextrins (CDs) and a linear axle polymer capped with bulky stopper molecules [1–3]. Because the threading CDs are associated along the polymer chain via non-covalent intermolecular interactions, they can move freely along the polymer chain. This free mobility of threading CDs in PRXs is attractive for the development of functional supramolecular materials [4–6]. In PRX applications, chemical modification of the threading CDs is important to reduce intermolecular interactions of the PRXs, impart solubility in organic solvents and water, and introduce functional molecules [7–9]. In this regard, various methods of introducing functional groups at the threading CD moieties of PRXs are used. Among the various chemical modifications, the introduction of azide or alkynyl groups at the threading CDs of the PRX is particularly attractive, because these functional groups undergo efficient azide–alkyne Huisgen [2 + 3] dipolar cycloaddition reactions, or click reactions [10–12]. Indeed, our group has reported on PRXs bearing azide groups at the threading CDs for the introduction of ligands and fluorescent molecules in biomaterials applications [13–14].

PRXs bearing reactive azide or alkynyl groups at the terminals of the axle polymers have an attractive designs for the fabrication of biomaterials, because the terminal azide or alkynyl groups in the PRXs can be utilized for the modification of other polymer chains and functional molecules. In general, PRXs comprising an inclusion complex of a linear polymer and threading CDs are typically synthesized via a two-step reaction: the preparation of pseudopolyrotaxanes, and the subsequent modification of the end groups of an axle polymer using bulky stopper molecules to prevent CD dethreading [1–3]. For the synthesis of end-reactive PRXs, the introduction of reactive functional groups during the preparation of PRXs is both simple and convenient. However, although the capping of pseudopolyrotaxanes via click reactions between azide (or alkyne)-terminated axle polymers and bulky stoppers containing alkyne (or azide) groups is employed broadly [15–21], few have reported on the synthesis of PRXs bearing terminal azides or alkynes [22–23]. This is probably because of the difficulties in synthesizing sufficient amounts of bulky stopper molecules containing azide or alkynyl groups for the capping reaction.

Herein, we describe a simple synthesis method for end-reactive PRXs using commercially available 4-substituted benzoic acids. Typically, 4-substituted benzoic acids are unsuitable for the capping of pseudopolyrotaxanes because they are insufficiently bulky. In this study, bis(2-amino-3-phenylpropyl) poly(ethylene glycol) (PEG-Ph-NH_2_) (**3**) was utilized as an axle polymer for the synthesis of end-reactive PRXs [24]. The terminal 2-amino-3-phenylpropyl groups in PEG-Ph-NH_2_ allow the formation of a pseudopolyrotaxane with α-CDs and simultaneously contribute to increasing the bulkiness of the terminal stopper groups after the capping reaction. This allows us to synthesize PRXs using small stopper molecules (e.g., 4-substituted benzoic acids) that cannot block the dethreading of α-CDs themselves. In this study, the synthesis and characterization of PRXs bearing azide groups and their reactivity against model alkynes via copper-catalyzed click reactions are performed. Additionally, fluorescence labeling of the PRX and intracellular fluorescence imaging were performed as an example application.

## Results and Discussion

### Synthesis of azide-terminated PRXs using bis(2-amino-3-phenylpropyl) poly(ethylene glycol) and 4-substituted benzoic acids

**Scheme 1:**
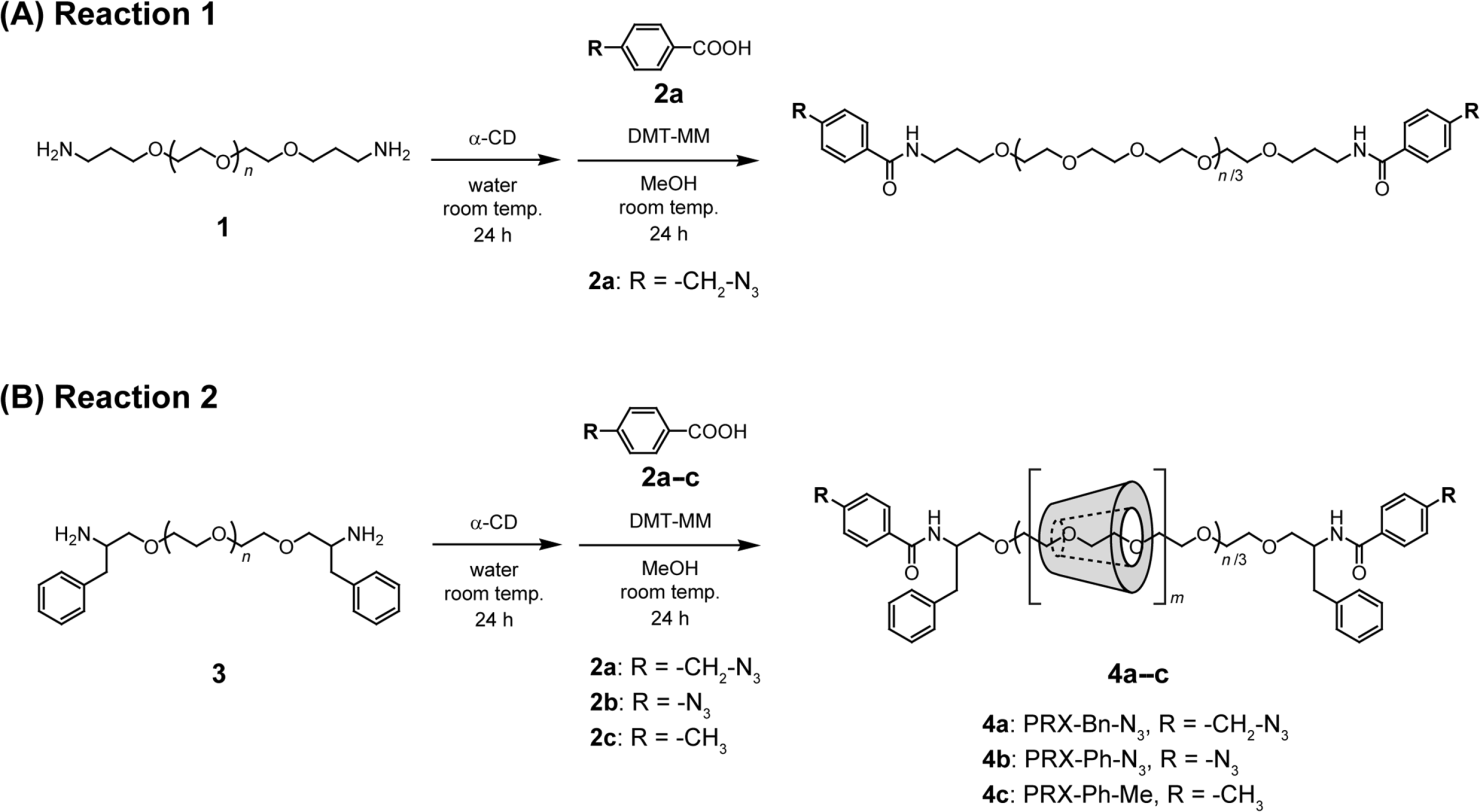
Scheme for the end-capping of the PEG-NH_2_ (**1**)/α-CD pseudopolyrotaxane with 4-(azidomethyl)benzoic acid (**2a**) (Reaction 1). Synthesis of end-reactive PRXs (**4a–c**) by the end-capping of the PEG-Ph-NH_2_ (**3**)/α-CD pseudopolyrotaxane with **2a–c** (Reaction 2).

To investigate whether PRXs could be obtained via 4-substituted benzoic acids, the end capping reaction of commonly utilized pseudopolyrotaxanes comprising bis(aminopropyl) PEG (PEG-NH_2_) (**1**) and α-CD was performed using 4-azidobenzoic acid as a model compound (Scheme 1A).

**Figure 1:**
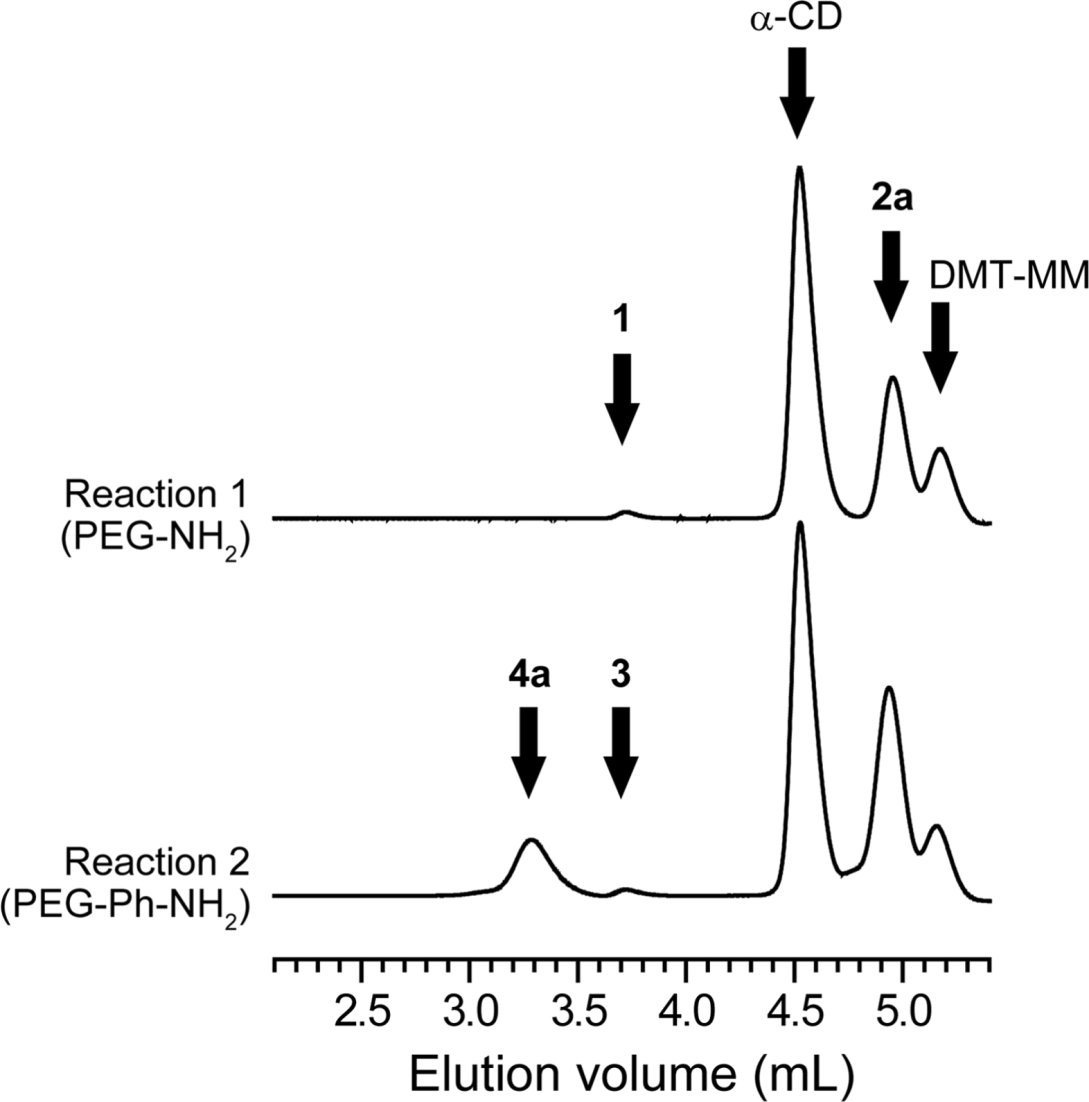
SEC charts for crude products of reaction 1 (upper chart) and 2 (lower chart).

The synthesis of PRXs was assessed with size exclusion chromatography (SEC) measurements in dimethyl sulfoxide (DMSO), in which the unreacted pseudopolyrotaxanes were readily dissociated into their constituent compounds. If PRXs were produced, the peak of the PRXs would be observed at an earlier elution volume than the PEG axle polymer (elution volume: 3.7 mL) or α-CD (elution volume: 4.5 mL) in the SEC chart. Figure 1 shows the SEC chart of the crude products of the PEG-NH_2_/α-CD pseudopolyrotaxane treated with 4-(azidomethyl)benzoic acid (**2a**) in the presence of 4-(4,6-dimethoxy-1,3,5-triazin-2-yl)-4-methylmorpholinium chloride (DMT-MM). However, the PRX peak is not detected, presumably because of the dethreading of α-CDs, even though the terminal groups of the axle polymer are capped with **2a**. Therefore, **2a** is insufficient bulky to prevent the dethreading of CDs from PRXs. To achieve the synthesis of end-reactive polyrotaxanes using 4-substituted benzoic acids as capping reagents, it is necessary to increase the bulkiness of the terminal group of the axle polymer.

In this regard, we next performed the capping reaction of pseudopolyrotaxanes composed of bis(2-amino-3-phenylpropyl) PEG (PEG-Ph-NH_2_) (**3**) as an axle polymer and threading α-CDs using the same 4-substituted benzoic acids (Scheme 1B). The reaction was performed in a heterogeneous system to prevent the dethreading of the α-CDs. The terminal 2-amino-3-phenylpropyl groups were introduced in **3** to increase the bulkiness after capping with **2a**. As a result of the capping reaction, the PRX peak is clearly observed at the elution volume of 3.3 mL in the SEC chart of the crude products (Figure 1). This result indicates that the successful synthesis of the end-reactive PRX (**4a**) was achieved by using **3** as an axle polymer. The yield of **4a** was determined to be 21.9% (calculated based on the molecular equivalents of the PEG axle). Many mechanisms can reduce the yield of PRXs including dethreading of CDs from the axle polymer in the process of the end capping reaction, the limited reaction efficiency of the stopper molecules in a heterogeneous system, and the loss of PRXs in the process of purification. Therefore, optimization of the reaction conditions and purification methods are required to increase the PRX yield.

**Figure 2:**
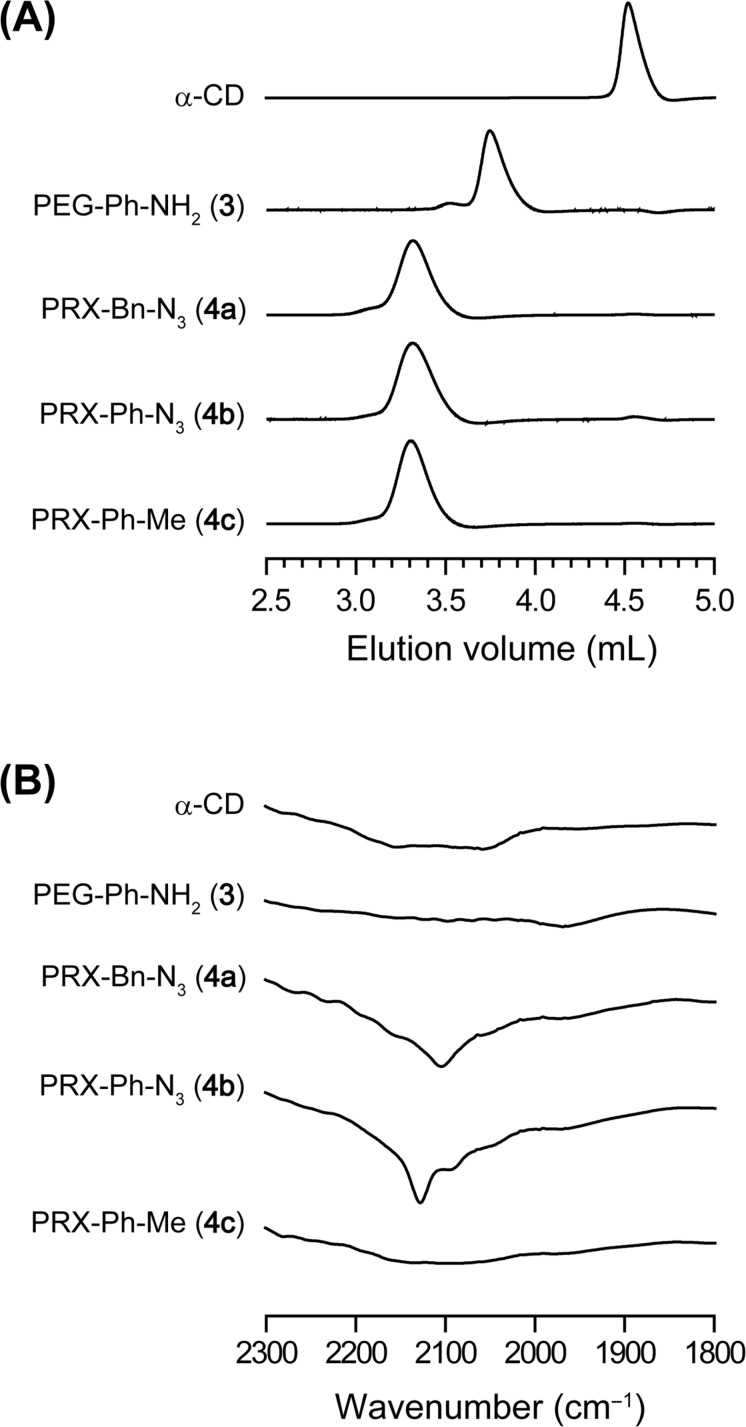
(A) SEC charts of α-CD, PEG-Ph-NH_2_ (**3**), PRX-Bn-N_3_ (**4a**), PRX-Ph-N_3_ (**4b**), and PRX-Ph-Me (**4c**). (B) FTIR transmission spectra of α-CD, PEG-Ph-NH_2_ (**3**), PRX-Bn-N_3_ (**4a**), PRX-Ph-N_3_ (**4b**), and PRX-Ph-Me (**4c**).

Next, to investigate whether other 4-substituted benzoic acids can be used as capping reagents for the preparation of the PEG-Ph-NH_2_/α-CD-based PRXs, the same capping reaction was demonstrated using 4-azidobenzoic acid (**2b**) and 4-methylbenzoic acid (**2c**). SEC charts of the resulting PRXs clearly demonstrated the successful synthesis of the PEG-Ph-NH_2_/α-CD-based PRXs (**4a–c**) using **2b** and **2c** (Figure 2A). The chemical composition of the resulting PRXs is summarized in Table 1. The number of threading α-CDs in **4a–c** is almost equal among the products, suggesting that the chemical composition of the obtained PRXs is not affected by the capping reagents. Additionally, **4a–c** exhibit narrow molecular weight distributions (Table 1) and negligible contamination by free α-CDs (Figure 2A). According to these results, **3** is an optimal axle polymer to synthesize PRXs with a variety of 4-substituted benzoic acids.

**Table 1:** Reaction conditions and characterizations of the PRXs.

Sample code	Feed [α-CD]/[PEG-Ph-NH_2_] molar ratio	Number of threading α-CDs^a^	*M*_n,NMR_^b^	*M*_w_/*M*_n_^c^

PRX-Bn-N_3_ (**4a**)	51.3	40.9 (36.7%)	50,100	1.12
PRX-Ph-N_3_ (**4b**)	51.3	42.8 (38.4%)	51,900	1.14
PRX-Ph-Me (**4c**)	51.3	41.5 (37.3%)	50,700	1.13

^a^Determined by ^1^H NMR in NaOD/D_2_O. The values in parentheses denote the percentage of α-CD coverage on the PEG chain, assuming one α-CD molecule includes two repeating units of ethylene glycol. ^b^Calculated based on the chemical composition of the PRXs determined by ^1^H NMR. ^c^Determined by SEC in DMSO containing 10 mM LiBr at 65 °C.

To confirm the introduction of reactive azide groups at the terminals of PRX-Bn-N_3_ (**4a**) and PRX-Ph-N_3_ (**4b**), Fourier-transform infrared (FTIR) spectra of the obtained PRXs were measured (Figure 2B, expanded views of FTIR spectra are shown in Supporting Information File 1, Figure S1). In the FTIR spectrum of **4a**, the asymmetric stretching mode of the terminal benzylazide groups is clearly observed at 2102 cm^−1^ [25–26]. For **4b**, the symmetric and asymmetric stretching modes of the terminal phenylazide groups are observed at 2127 and 2092 cm^−1^, respectively [27]. PRX-Ph-Me (**4c**) exhibits negligible peaks in this region. In addition, proton nuclear magnetic resonance (^1^H NMR) spectra of **4a–c** are well characterized by the chemical structures of the products (Supporting Information File 1, Figure S2). These results clearly demonstrate the successful synthesis of PRXs with well-defined terminal structures.

### Reactivity of terminal azide groups in PRXs with alkynes via copper-catalyzed click reaction

**Scheme 2:**
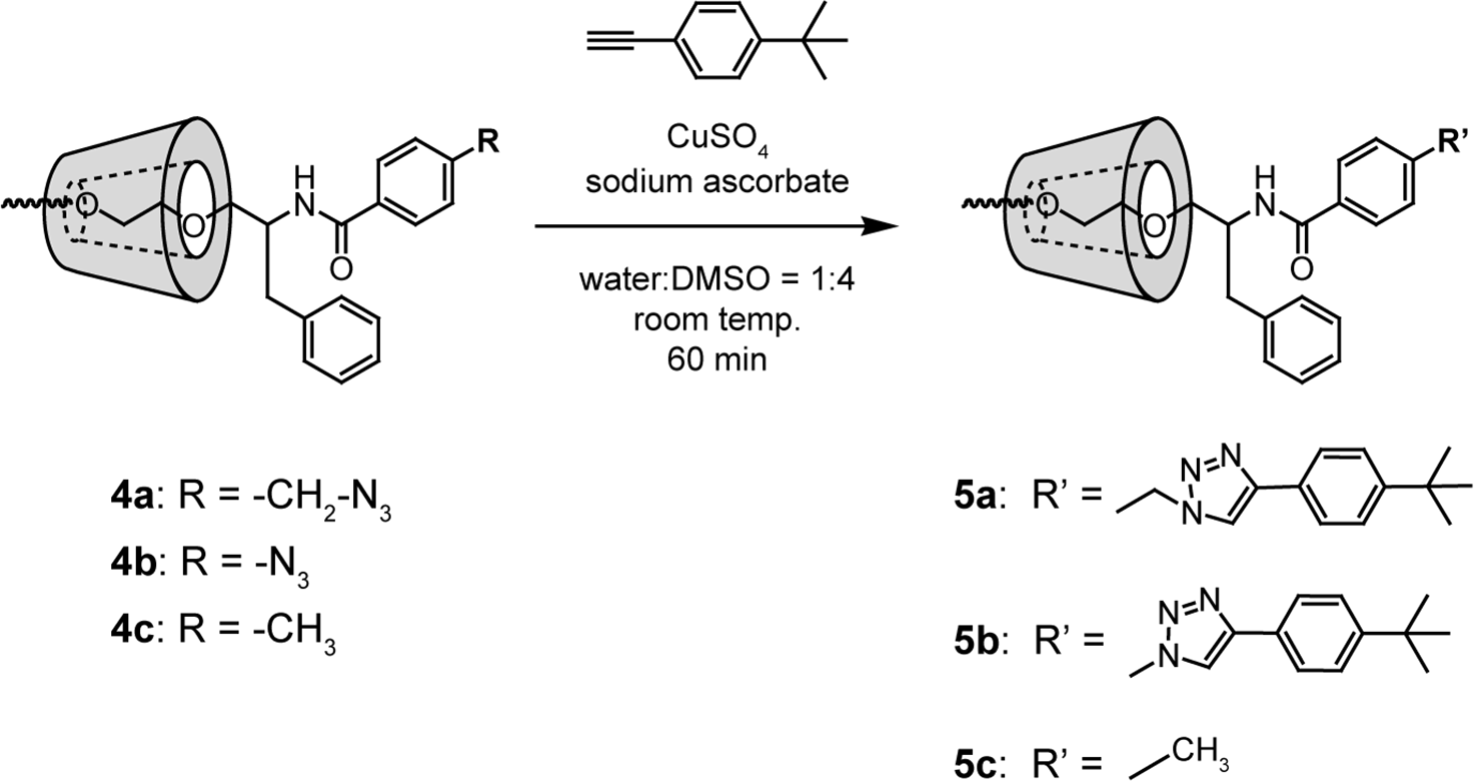
End-group modification of **4a-c** with model alkyne via copper-catalyzed click reaction.

**Figure 3:**
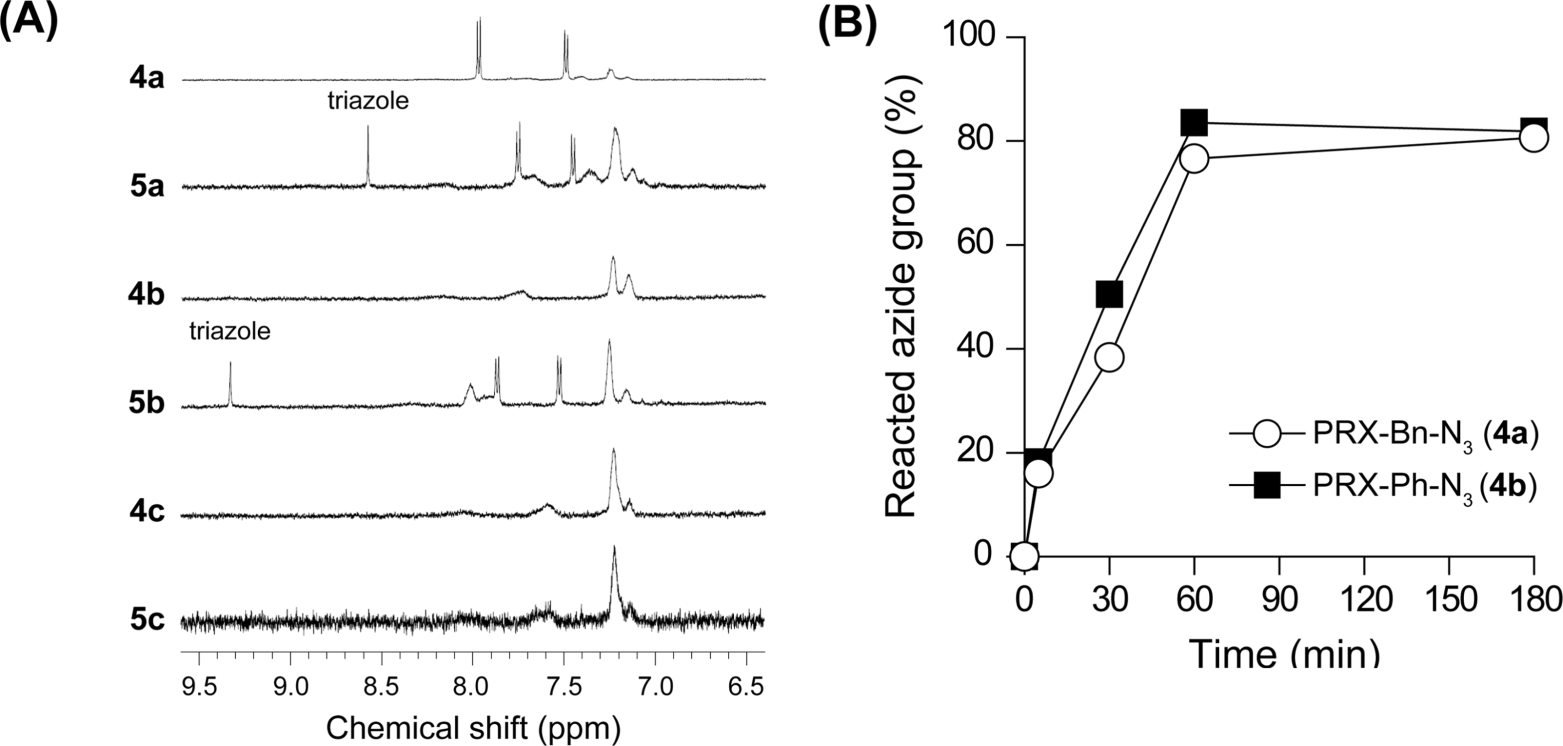
(A) ^1^H NMR spectra of the PRXs before (**4a**, **4b**, **4c**) and after copper-catalyzed click reactions with *p*-(*tert*-butyl)phenylacetylene for 60 min (**5a**, **5b**, **5c**). Spectra were recorded in DMSO-*d*_6_. (B) Time-course of click reaction between the azide-terminated PRXs (**4a**: open circles, **4b**: closed squares) and *p*-(*tert*-butyl)phenylacetylene.

The reactivity of the terminal azide groups of **4a** and **4b** with alkynes via the copper-catalyzed click reaction was examined. In this experiment, *p*-(*tert*-butyl)phenylacetylene was utilized as the model alkyne. One equivalent of *p*-(*tert*-butyl)phenylacetylene was allowed to react with the terminal azide groups of the PRXs in the presence of CuSO_4_ and sodium ascorbate (Scheme 2). After 60 min of reaction, ^1^H NMR spectra of the obtained products (**5a–c**) were measured (Figure 3A). In the ^1^H NMR spectra of **5a** and **5b**, the protons assignable to 1,2,3-triazole linkages are observed at 8.5 and 9.4 ppm, respectively, which were consistent with the previous report [28]. These results suggest that the copper-catalyzed click reaction with *p*-(*tert*-butyl)phenylacetylene proceeds for both **4a** and **4b**. However, the ^1^H NMR spectra of **4c** and **5c** are almost identical, despite the click reaction being conducted. This result indicates that the copper-catalyzed click reaction with *p*-(*tert*-butyl)phenylacetylene is selective for the terminal azide groups of **4a** and **4b**.

Next, the kinetics for the reaction between **4a**,**b** and *p*-(*tert*-butyl)phenylacetylene was investigated. Briefly, 1 mol equivalent of *p*-(*tert*-butyl)phenylacetylene was allowed to react with the terminal azide groups of the PRXs. The ratio of terminal azide groups in the PRXs reacted with *p*-(*tert*-butyl)phenylacetylene molecules was determined by ^1^H NMR. This reveals that the copper-catalyzed click reaction between azide-terminated **4a**,**b** and *p*-(*tert*-butyl)phenylacetylene proceeds rapidly, reaching a plateau value within 60 min (Figure 3B). In addition, approximately 80% of azide groups reacted with *p*-(*tert*-butyl)phenylacetylene after 60 min, suggesting that the click reaction with the terminal azide groups of **4a,b** occurred quickly and efficiently. In general, aliphatic azide groups (i.e*.*, benzylazide) are more reactive than aromatic azide groups (i.e*.*, phenylazide) in the click reaction with alkynes [28]. In our experiments, **4a** and **4b** exhibited similar reaction efficiencies with *p*-(*tert*-butyl)phenylacetylene, but this may change with the reaction conditions and alkyne species.

### End-group functionalization of PRXs for fluorescence imaging

**Scheme 3:**
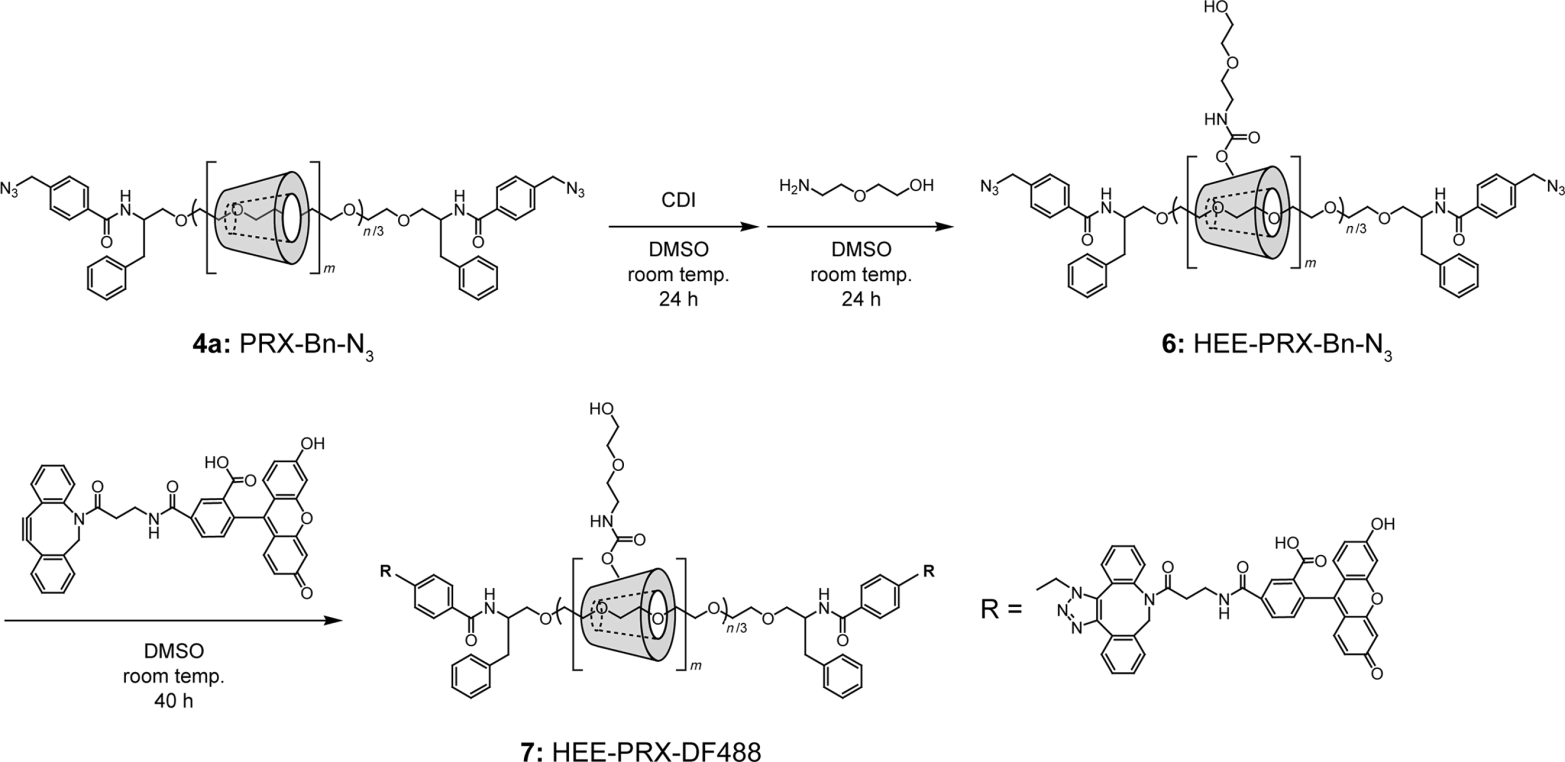
Scheme of the synthesis of water-soluble PRX (**6**) and the following end group modification with copper-free click reaction (**7**).

Terminal reactive groups in the PRXs can be utilized in various biomaterials applications, such as the fabrication of cross-linked materials (e.g., hydrogels) [29], direct surface immobilization onto alkyne-immobilized surfaces [30], and the modification of other functional molecules for drug delivery [31]. Herein, alkynyl group-bearing fluorescent molecules were modified at the terminal azide group of the PRXs, and intracellular fluorescence imaging of the PRXs was performed to verify whether the terminally modified fluorescent molecules could be used for fluorescence imaging. First, **4a** was modified with 2-(2-hydroxyethoxy)ethyl (HEE) groups to impart water solubility, which was necessary for in vitro cellular experiments (Scheme 3) [32–33]. Then, the terminal benzylazide groups of HEE-PRX-Bn-N_3_ (**6**) were modified with dibenzylcyclooctyne (DBCO)-conjugated fluorescent molecules (DF488) via a copper-free click reaction (Scheme 3) [34–35].

**Figure 4:**
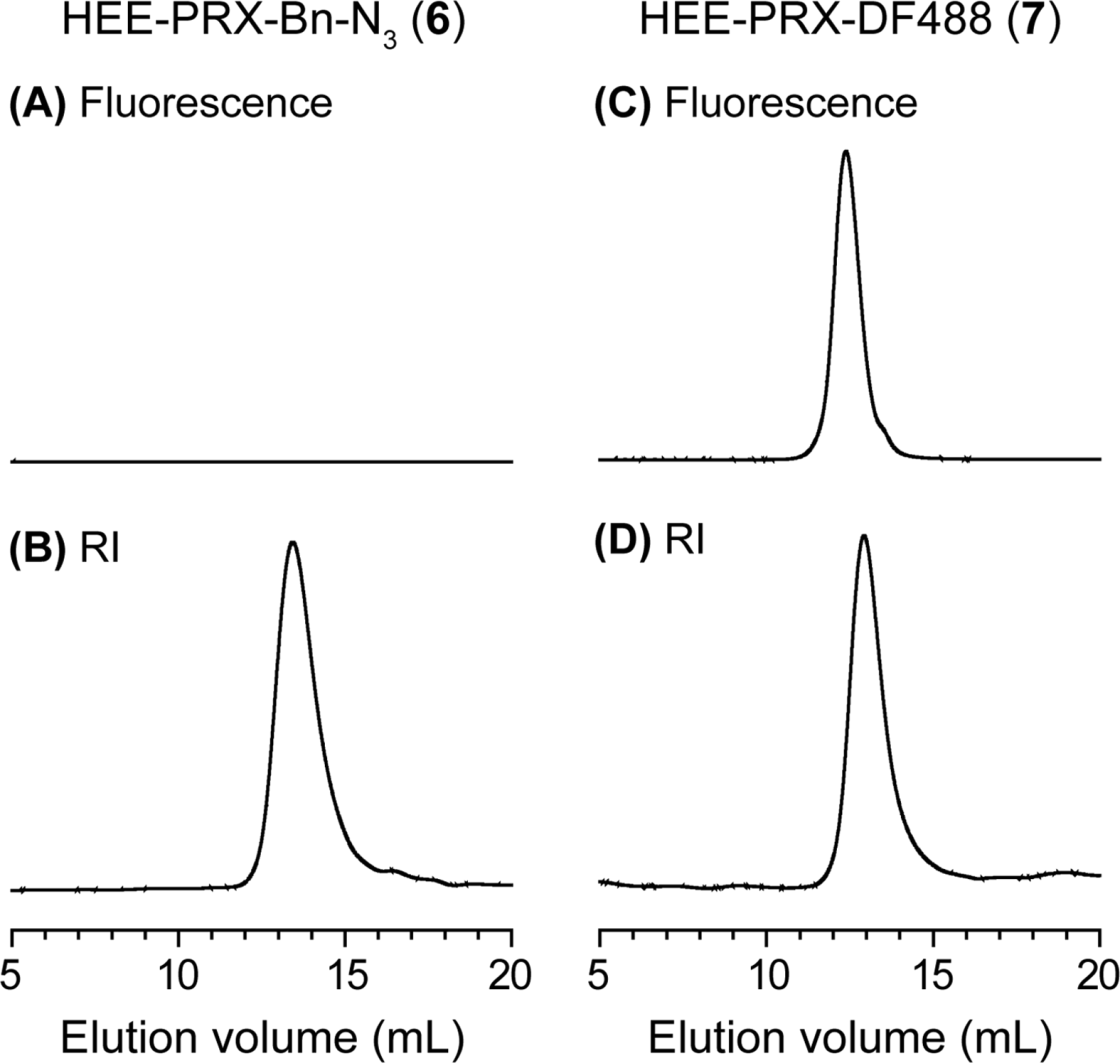
SEC charts of the HEE-PRX-Bn-N_3_ (**6**) (A, B) and the HEE-PRX-DF488 (**7**) (C, D) monitored with fluorescence detector (excitation wavelength: 488 nm, emission wavelength: 515 nm) (A, C) and refractive index (RI) detector (B, D).

After the modification, the introduction of DF488 at the terminal of **6** is confirmed by SEC measurements equipped with a fluorescence detector (Figure 4). Compound **6** exhibits negligible peaks in fluorescence detection, whereas a unimodal peak is observed in refractive index detection. Meanwhile, HEE-PRX-DF488 (**7**) exhibits a unimodal peak in both fluorescence and refractive index detection, clearly indicating the successful modification of the fluorescent molecules via the copper-free click reaction. Additionally, the terminal azide groups in **6** were almost entirely modified with fluorescent molecules.

**Figure 5:**
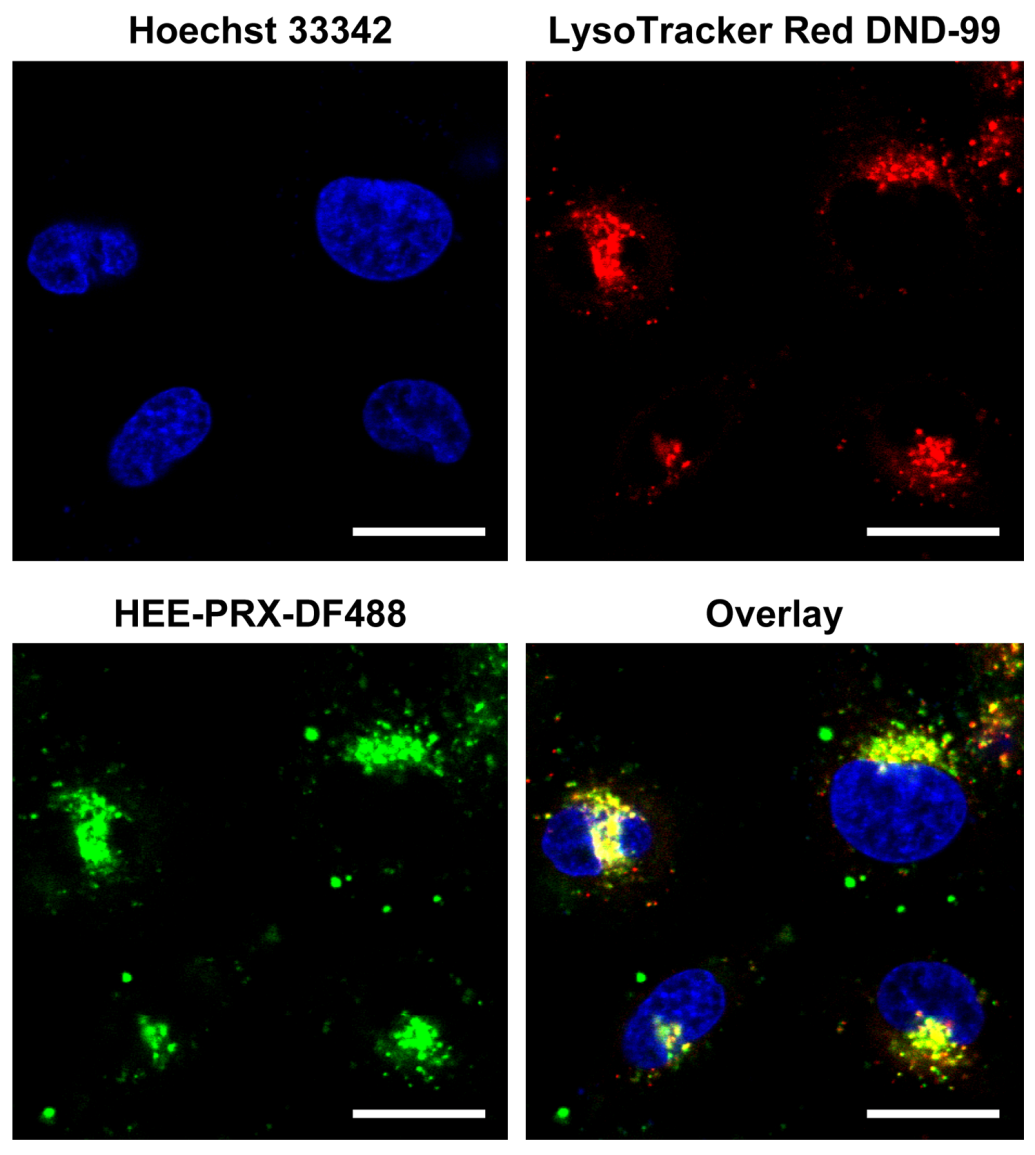
CLSM images of HeLa cells treated with HEE-PRX-DF488 (**7**) (500 μg/mL) for 26 h (scale bars: 20 μm). The acidic endosomes/lysosomes and nuclei are stained with LysoTracker Red DND-99 and Hoechst 33342, respectively.

In our previous study relating to drug delivery applications of PRXs, fluorescent molecules were modified on the threading α-CDs in the PRXs to monitor intracellular uptake and localization of the PRXs [14,32,36–37]. To verify whether **7** could be utilized for monitoring the intracellular internalization of the PRXs, the intracellular uptake of **7** was investigated. HeLa cells were treated with **7** for 26 h, before observation by confocal laser scanning microscopy (CLSM) (Figure 5). The punctate **7** is clearly observed at the perinuclear region of the HeLa cells. The **7** puncta is highly co-localized with the LysoTracker Red, indicating that **7** was internalized into the cells via endocytosis and localized in acidic endosomes and lysosomes. This intracellular uptake pathway and localization of **7** is consistent with our previous reports [14,32,36–37]. Accordingly, we concluded that the terminal azide groups in **4a** can be utilized as installation moieties for fluorescent molecules to monitor intracellular fate of PRXs. Although the number of installation sites in the azide-terminated PRXs is limited to two, it is considered that the installation of two fluorescent molecules at the terminals of the PRX is sufficient for detecting the intracellular trafficking and biodistribution of PRXs by fluorescence microscopy [38]. In addition, the terminal azide groups in the PRXs can act as installation moieties for magnetic resonance imaging (MRI) contrast agents and positron emission tomography (PET) probes for in vivo studies.

## Conclusion

In this study, we described a novel preparation method for end-reactive PRXs using **3** as an axle polymer for the PRXs. The terminal 2-amino-3-phenylpropyl groups of **3** prevent the dethreading of α-CDs after end-capping with 4-substituted benzoic acids such as **2a** and **2b**. The terminal azide groups of **4a** and **4b** exhibited fast and efficient reactions with *p*-(*tert*-butyl)phenylacetylene via a copper-catalyzed click reaction. In addition, the terminal azide groups of **4a** were modified with DBCO-conjugated fluorescent molecules via a copper-free click reaction. Finally, successful intracellular fluorescence imaging was achieved using the fluorescently labeled compound **7**. In addition to such fluorescent molecules, many functional molecules could be introduced at the terminal of the PRXs via copper-catalyzed or copper-free click reactions, including ligand molecules for active targeting, radioactive molecules for in vivo imaging, hydrophobic polymers for the surface immobilization of PRXs, and hydrophilic polymers for acquiring water solubility. Accordingly, the azide group-terminated end-reactive **4a** and **4b** are expected to be useful candidates in the fabrication or functionalization of supramolecular biomaterials, such as cross-linked hydrogels, surface immobilization, and drug delivery carriers for biomolecules [6]. Further studies relating to the biomaterials applications of azide group-terminated PRXs are currently underway in our laboratory and will be reported elsewhere.

## Experimental

### Materials

α,ω-Bis(2-amino-3-phenylpropyl) poly(ethylene glycol) (PEG-Ph-NH_2_) (*M*_n_ = 10,060, *M*_w_/*M*_n_ = 1.02) was synthesized according to our previous report [24]. α,ω-Bisaminopropyl PEG (PEG-NH_2_) (*M*_n_ = 10,200, *M*_w_/*M*_n_ = 1.03) was obtained from NOF Corporation (Tokyo, Japan). α-Cyclodextrin (α-CD) was obtained from Ensuiko Sugar Refining (Tokyo, Japan). 4-(Azidomethyl)benzoic acid was synthesized according to the previous report [27]. 4-Azidobenzoic acid, 4-methylbenzoic acid, *p*-(*tert*-butyl)phenylacetylene, and 2-(2-hydroxyethoxy)ethylamine (HEEA) were obtained from TCI (Tokyo, Japan). 4-(4,6-Dimethoxy-1,3,5-triazin-2-yl)-4-methylmorpholinium chloride (DMT-MM) and copper(II) sulfate pentahydrate (CuSO_4_) were obtained from Wako Pure Chemical Industries (Osaka, Japan). *N*,*N*’-Carbonyldiimidazole (CDI) and (+)-sodium L-ascorbate were obtained from Sigma-Aldrich (St. Louis, MO, USA). Dibenzocyclooctyne-fluor 488 (DF488) was obtained from Click Chemistry Tools (Scottsdale, AZ, USA). Other solvents were obtained from Kanto Chemicals (Tokyo, Japan).

### Characterization of PRXs

SEC was performed out on an HLC-8120 system (Tosoh, Tokyo, Japan) equipped with a combination of TSKgel AW-4000 and AW-2500 columns (150 mm × 6 mm ID) (Tosoh), eluted with dimethylsulfoxide (DMSO) containing 10 mM LiBr at a flow rate of 0.15 mL/min at 65 °C. The polydispersity index (*M*_w_/*M*_n_) was calculated from a calibration curve of standard PEGs (Agilent Technologies, Wilmington, DE, USA). For SEC with fluorescence detection, the measurements were performed on a Gulliver system (Jasco, Tokyo, Japan) consisting of a DG-2080–53 degasser (Jasco), a PU-980 pump (Jasco), an AU-950 autosampler (Jasco), a CO-965 column oven (Jasco), an FP-920 fluorescence detector (excitation wavelength: 488 nm, emission wavelength: 515 nm) (Jasco), an RI-2031 Plus refractive index detector (Jasco), and a combination of TSKgel α-4000 and TSKgel α-2500 columns (300 mm × 7.8 mm ID) (Tosoh). The solutions (50 μL) were injected into the SEC system, and the system was eluted with a mixture of water and DMSO (volume ratio 50:50) at a flow rate of 0.3 mL/min at 40 °C. ^1^H NMR spectra were recorded on a Bruker Avance III 500 MHz spectrometer (Bruker BioSpin, Rheinstetten, Germany). FTIR spectra were recorded on a Spectrum 100 FTIR spectrometer (Perkin Elmer, Wellesley, MA, USA). The sample powder was mixed with KBr and pellets were prepared for FTIR measurements.

#### Synthesis of benzylazide group-terminated PRX (**4a**)

PEG-Ph-NH_2_ (300 mg, 29.9 μmol) dissolved in a small aliquot of water was added to the α-CD aqueous solution (10.3 mL, 145 mg/mL), and the mixture was stirred for 24 h at room temperature. After the reaction, the precipitate was collected by centrifugation and freeze-dried for 1 day to obtain a pseudopolyrotaxane as powder (yield 1.37 g). Then, 4-(azidomethyl)benzoic acid (106 mg, 597 μmol), DMT-MM (165 mg, 597 μmol), and the pseudopolyrotaxane were allowed to react in methanol (14 mL) for 24 h at room temperature. The precipitate was collected by centrifugation and washed three times with methanol, and then dissolved in DMSO and reprecipitated into water. This reprecipitation process was repeated until all free α-CD was removed. The recovered precipitate was dispersed into water and freeze-dried to obtain a benzylazide group-terminated PRX (**4a**) (327.9 mg, 21.9% yield based on PEG mol %). The number of threading α-CDs in **4a** was determined by ^1^H NMR in DMSO-*d*_6_. ^1^H NMR (500 MHz, D_2_O) δ 3.2–4.1 (m, PEG backbone and H_2_, H_3_, H_4_, H_5_, and H_6_ protons of α-CD), 4.43 (s, -C*H_2_*- of benzylazide), 4.95 (m, H_1_ proton of α-CD), 7.2–7.3 (m, aromatics of benzyl group), 7.42 (d, aromatics of benzylazide), 7.59 (d, aromatics of benzylazide).

#### Synthesis of phenylazide group-terminated PRX (**4b**)

The PRXs capped with 4-azidobenzoic acid were synthesized as described above (48.8% yield based on PEG mol %). ^1^H NMR (500 MHz, D_2_O) δ 3.2–3.8 (m, PEG backbone and H_2_, H_3_, H_4_, H_5_, and H_6_ protons of α-CD), 4.95 (m, H_1_ proton of α-CD), 7.04 (d, aromatics of phenylazide), 7.1–7.3 (m, aromatics of benzyl group), 7.54 (d, aromatics of phenylazide).

#### Synthesis of 4-methylphenyl group-terminated PRX (**4c**)

The PRXs capped with 4-methylbenzoic acid were synthesized as described above (48.5% yield based on PEG mol %). ^1^H NMR (500 MHz, D_2_O) δ 2.28 (m, -C*H_3_* of methylphenyl group), 3.2–4.1 (m, PEG backbone and H_2_, H_3_, H_4_, H_5_, and H_6_ protons of α-CD), 4.95 (m, H_1_ proton of α-CD), 7.1–7.3 (m, aromatics of benzyl group and methylphenyl group), 7.43 (d, aromatics of methylphenyl group).

#### Reactivity of terminal azide groups in PRXs with alkynes via copper-catalyzed click reaction (**5a**, **5b**)

The typical procedure for investigating the reactivity of **4a** was as follows: **4a** (122.2 mg, 2.31 μmol of PRX, 4.62 mM of azide groups) and *p*-(*tert*-butyl)phenylacetylene (0.82 μL, 4.62 μmol, 1 mol equivalent to azide group in **4a**) were dissolved in DMSO (6.4 mL). Then, CuSO_4_ (16.1 mg, 81.1 μmol) and sodium ascorbate (40.2 mg, 161 μmol) were dissolved in water (1.6 mL) and added to the reaction mixture (the concentration of CuSO_4_ and sodium ascorbate in the reaction mixture were 10.1 mM and 20.1 mM, respectively). The reaction mixture was allowed to stir at room temperature. At prescribed time periods, an aliquot of the reaction mixture was collected (2 mL) and purified by dialysis against water for three days (molecular weight cut-off of 3500; Fast Gene, Nippon Genetics, Tokyo, Japan). The recovered solutions were freeze-dried to obtain the PRXs as powders. The ratio of terminal azide groups in **4a** reacted with *p*-(*tert*-butyl)phenylacetylene was calculated from the ^1^H NMR peak area between 1.3 ppm (-C(C*H_3_*) of *p*-(*tert*-butyl)phenylacetylene) and 4.8 ppm (H_1_ proton of α-CD threading onto **4a**). ^1^H NMR (500 MHz, DMSO-*d*_6_) δ 1.29 (-C(C*H_3_*)_3_ of *tert*-butylphenyl group), 3.2–3.8 (m, PEG backbone and H_2_, H_3_, H_4_, H_5_, and H_6_ protons of α-CD), 4.45 (m, O_6_*H* of α-CD), 4.80 (m, H_1_ of α-CD), 5.49 (m, O_3_*H* of α-CD), 5.65 (m, O_2_*H* of α-CD), 7.1–7.3 (m, aromatics derived from 2-amino-3-phenylpropyl group), 7.36 (br, aromatics derived from 4-(azidomethyl)benzoic acid), 7.46 (d, aromatics of *tert*-butylphenyl group), 7.68 (br, aromatics derived from 4-(azidomethyl)benzoic acid), 7.76 (d, aromatics of *tert*-butylphenyl group), 8.53 (s, triazole).

The same reaction was performed for **4b** and **4c** according to the above described procedure.

#### Synthesis of water-soluble benzylazide group-terminated PRX (**6**)

To solubilize **4a** into an aqueous solutions, 2-(2-hydroxyethoxy)ethyl (HEE) groups were modified onto the threading α-CDs of **4a** [28–29]. Briefly, **4a** (100 mg, 2 μmol of PRX, 81.6 μmol of α-CD) and CDI (132 mg, 816 μmol) were dissolved in dehydrated DMSO (5 mL), and the solution was stirred for 24 h at room temperature under a nitrogen atmosphere. Then, HEEA (81 μL, 816 μmol) was added to the reaction mixture and the solution was stirred for an additional 24 h at room temperature under a nitrogen atmosphere. After the reaction, the PRX was purified by dialysis against water for three days (molecular weight cut-off of 12,000–14,000, Fast Gene, Nippon Genetics). The recovered solution was freeze-dried to obtain **6** as powder (117.7 mg, 73.8% yield). The number of HEE groups in **6** was determined to be 226.6 by comparing the ^1^H NMR peak area between 3.12 ppm (-NH-C*H_2_*-CH_2_-O- of HEE group) and 4.7–5.2 ppm (H_1_ proton of α-CD). The *M*_n_ of **6** was determined to be 79,800 from the ^1^H NMR. ^1^H NMR (500 MHz, DMSO-*d*_6_) δ 3.12 (m, -NH-C*H_2_*-CH_2_- of HEE group), 3.2–4.5 (m, PEG backbone and H_2_, H_3_, H_4_, H_5_, and H_6_ protons of α-CD), 4.57 (m, O_6_*H* of α-CD), 4.84 (m, H_1_ of α-CD), 5.60 (m, O_2_*H* and O_3_*H* of α-CD), 7.04 (m, -N*H*-CH_2_-CH_2_- of HEE group), 6.9–7.3 (m, aromatics derived from 2-amino-3-phenylpropyl group and 4-(azidomethyl)benzoic acid).

#### Terminal modification of **6** via copper-free click reaction

**6** (46.2 mg, 576 nmol of PRX, 1.15 μmol of azide groups) and DF488 (1.0 mg, 6.36 μmol, 5.5 mol equivalent to azide group in the HEE-PRX-Bn-N_3_) were dissolved in DMSO (4 mL) and the reaction mixture was allowed to stir for 40 h at room temperature. Then, the PRX was purified by dialysis against water for three days (molecular weight cut-off of 10,000, Thermo Fisher Scientific, Waltham, MA, USA). The recovered solutions were freeze-dried to obtain HEE-PRX-DF488 (**7**) as a yellow powder (43.2 mg, 92.0% yield). The degree of DF488 in the terminals of **7** was determined to be 96.1% by ^1^H NMR spectroscopy. ^1^H NMR (500 MHz, DMSO-*d*_6_) δ 3.14 (m, -NH-C*H_2_*-CH_2_- of HEE group), 3.2–4.4 (m, PEG backbone and H_2_, H_3_, H_4_, H_5_, and H_6_ protons of α-CD), 4.58 (m, O_6_*H* of α-CD), 4.85 (m, H_1_ of α-CD), 5.63 (m, O_2_*H* and O_3_*H* of α-CD), 7.05 (m, -N*H*-CH_2_-CH_2_- of HEE group), 6.4-8.8 (m, aromatics derived from 2-amino-3-phenylpropyl group, 4-(azidomethyl)benzoic acid, and DF488).

### Cellular internalization

HeLa cells derived from human cervical carcinoma were obtained from the Japanese Collection of Research Bioresources (JCRB, Osaka, Japan). The cells were cultured in Dulbecco’s modified Eagle’s medium (DMEM) (Wako Pure Chemical Industries) containing 10% fetal bovine serum (FBS) (Sigma-Aldrich), 100 units/mL penicillin, and 100 µg/mL streptomycin (Wako Pure Chemical Industries) in a humidified 5% CO_2_ atmosphere at 37 °C. HeLa cells were plated on 35 mm glass-bottom dishes (Iwaki, Tokyo, Japan) at a density of 1 × 10^4^ cells/dish and incubated overnight. After the medium was exchanged with fresh DMEM (135 μL), the cells were treated with **7** (15 µL) (concentration: 500 μg/mL) for 26 h. Then, the cells were stained with LysoTracker Red DND-99 (Thermo Fisher Scientific) (500 nM) for 30 min, followed by staining with Hoechst 33342 (Dojindo Laboratories, Kumamoto, Japan) (1 μg/mL) for 10 min at 37 °C. CLSM images were acquired with a FluoView FV10i (Olympus, Tokyo, Japan) equipped with a 60x water-immersion objective lens (N/A 1.2) and a diode laser.

## Supporting Information

File 1 FTIR and ^1^H NMR spectra of the PRXs.
