# Supplementary material for: Versatile synthesis of end-reactive polyrotaxanes applicable to fabrication of supramolecular biomaterials
Source: Beilstein J Org Chem. 2016 Dec 28;12:2883–92. doi: 10.3762/bjoc.12.287 (PMC5238546; doi:10.3762/bjoc.12.287)
Supplement: File 1 — FTIR and 1H NMR spectra of the PRXs. [file Beilstein_J_Org_Chem-12-2883-s001.pdf]

## Supporting Information

**for**

# Versatile synthesis of end-reactive polyrotaxanes applicable to fabrication of supramolecular biomaterials

Atsushi Tamura, Asato Tonegawa, Yoshinori Arisaka, and Nobuhiko Yui*

Address: Department of Organic Biomaterials, Institute of Biomaterials and Bioengineering, Tokyo Medical and Dental University, 2-3-10 Kanda-Surugadai, Chiyoda, Tokyo 101-0062, Japan

Email: Nobuhiko Yui - yui.org@tmd.ac.jp

*Corresponding author

## FTIR and $^1$H NMR spectra of the PRXs

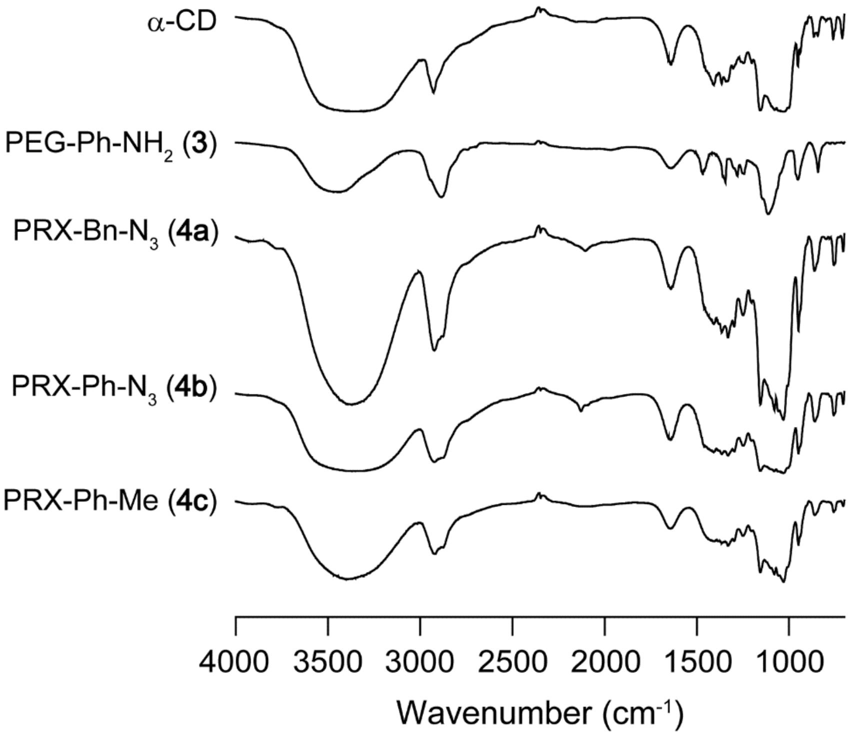


**Figure S1:** FTIR spectra of α-CD, PEG-Ph-$NH_2$ (**3**), PRX-Bn-$N_3$ (**4a**), PRX-Ph-$N_3$ (**4b**), and PRX-Ph-Me (**4c**).

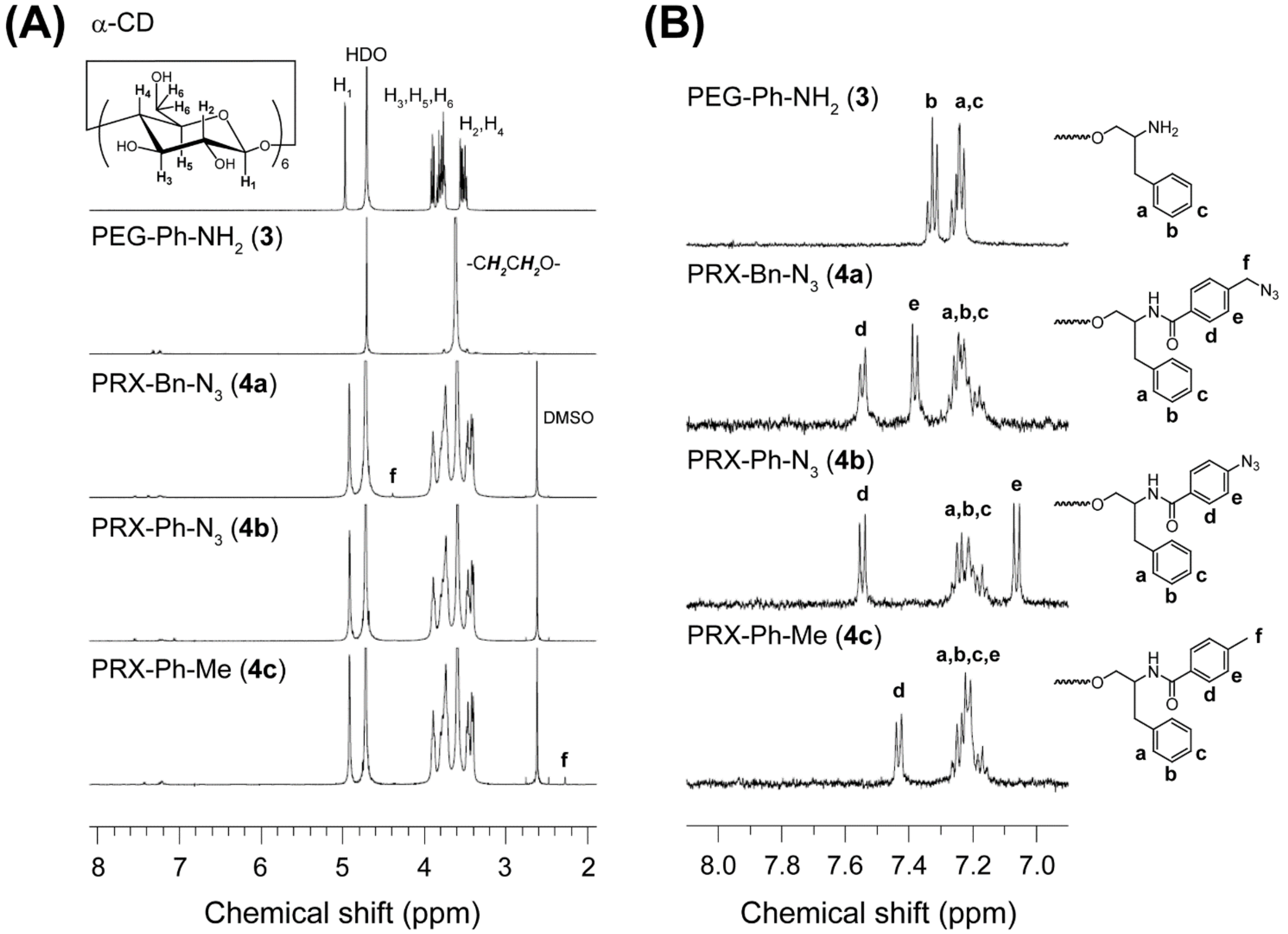


**Figure S2:** (A) $^{1}$H NMR spectra of α-CD, PEG-Ph-$NH_2$ (**3**), PRX-Bn-$N_3$ (**4a**), PRX-Ph-$N_3$ (**4b**), and PRX-Ph-Me (**4c**) in NaOD/$D_2O$. (B) Enlarged views of $^{1}$H NMR spectra of PEG-Ph-$NH_2$ (**3**), PRX-Bn-$N_3$ (**4a**), PRX-Ph-$N_3$ (**4b**), and PRX-Ph-Me (**4c**) in NaOD/$D_2O$.
